# The Effects of Surface Stiffness on Human Hopping Frequency Preference and the Underlying Neuromuscular Function of the Foot and Ankle

**DOI:** 10.1111/sms.70067

**Published:** 2025-05-20

**Authors:** Jonathon V. Birch, Luke A. Kelly, Dominic J. Farris

**Affiliations:** ^1^ Public Health & Sport Sciences University of Exeter Exeter Devon UK; ^2^ Human Movement and Nutrition Sciences The University of Queensland Brisbane Queensland Australia; ^3^ School of Health Sciences and Social Work Griffith University Gold Coast Queensland Australia

**Keywords:** bouncing, EMG, midfoot, plantar intrinsic muscles, surface compliance

## Abstract

Typically, humans tune their lower limb mechanics to preserve center of mass motion when hopping or running on surfaces with different stiffnesses. However, much of our understanding of this interaction is based on frequency‐constrained hopping and not preferred behavior, which may also be influenced by the stiffness of the surface underfoot. Therefore, we tested if preferred hopping frequency was different from a previously assumed value of 2.2 Hz and if preference was affected by a less‐stiff surface. To help explain any observed trends, we quantified foot and ankle mechanics and muscle activations for frequencies ±20% of preferred. We used custom‐built platforms to provide both an elastic and locked (inelastic) surface and asked participants to hop bilaterally in place on each. We measured multi‐segment foot and ankle kinematics and ground reaction forces, alongside electromyography (EMG) of flexor digitorum brevis, abductor hallucis, soleus, and tibialis anterior. There was no significant difference between mean preferred hopping frequency and 2.2 Hz, for either surface. There was also no difference in mechanics between preferred frequency and 2.2 Hz conditions. However, there were effects of surface, frequency, and surface‐by‐frequency interactions on foot and ankle kinematics, kinetics, and EMG. Frequency preference appears to be partially driven by an effort to maximize energy stored and returned in the surface while trading off the costs of active muscular work and the cost associated with producing force. Frequency affects hopping mechanics differently on stiff vs. elastic surfaces.

## 1 Introduction

To walk and run in the real world, humans are required to continually adapt our gait mechanics to ever‐varying conditions. The human proficiency for bipedalism is such that we generally adapt in a way that minimizes energy expenditure for those conditions [1, 2, 3]. For example, our environment is characterized by many substrates, each placing varied mechanical demands on the person. Any surface that is not infinitely stiff will deform, storing, returning, and dissipating energy. When faced with changes in surface stiffness in isolation, humans tune the combined stiffness of their lower limbs so that the oscillation of their body's center of mass (COM) remains in phase with the compression and recoil of the surface [4, 5, 6, 7]. As the energy returned from underfoot surfaces increases, hopping humans reduce active muscle work [8] and muscle activation of the ankle plantar flexors and intrinsic foot muscles [9]. This work and activation‐reducing strategy involves humans modifying their landing mechanics to allow them to harness elastic strain energy stored by the surface. This neuromuscular adaptation to spring‐loaded assistance is similar to that seen in response to wearable assistive exoskeletons whose springs store and return energy in parallel with skeletal joints [10, 11].

Humans often modify movement frequency of periodic gait mechanics to adapt to their environment. The relevance of movement frequency to adaptation of limb stiffness has been recognized by several authors and often studied using hopping movements as a simplified representation of the “bouncing” gait mechanics that characterize running [5, 12]. With increasing hopping cycle frequency and running stride frequency, leg stiffness increases allowing greater peak forces to be applied to the ground [12]. Therefore, frequency adjustments might be linked to lower limb stiffening that has been shown in response to reduced surface stiffness [4]. It is assumed that unconstrained humans will self‐select a preferred frequency at which energy requirements of the task are minimized while spring‐like mechanics are maintained [12]. However, it remains to be tested if movement frequency is preferentially altered in response to surfaces of different stiffness. It has often been accepted that 2.2 Hz represents the optimal frequency for human hopping and therefore others have tested the effects of surface stiffness at a controlled hopping frequency of 2.2 Hz [4, 12, 13]. However, this is informed by an analysis of only four participants on an infinitely stiff surface [12], and optimal frequencies are potentially influenced by changes in surface stiffness. For example, humans select longer step lengths and ground contact times when wearing cushioned shoes or running on a tuned running track than they do when unshod or running on an infinitely stiff surface [8, 14]. Therefore, our current understanding of how surface stiffness affects lower limb mechanics might be based on unfounded assumptions regarding preferred frequency. To fully understand how humans adapt bouncing gaits to changes in underfoot surface stiffness, it is necessary to first test if frequency preference is altered with changes in surface stiffness.

Furthermore, to then understand what mechanisms might underpin an interaction between frequency preference and surface stiffness, the associated neuromechanical adaptations should be considered. When exploring the mechanical alterations to hopping frequency at a joint level, it appears that both our ankles and feet are responsible for modulating the stiffness requirements of the lower limb [4, 7, 15]. Additionally, it has been shown that foot and ankle muscles adjust their activation levels to modify mechanical work in response to changes in surface stiffness [9]. However, it is not known how foot and ankle neuromechanics are adapted to concurrent changes in surface stiffness and hopping frequency.

Therefore, the aims of this study were twofold. We first sought to test if preferred human hopping frequency is affected by the mechanical stiffness of the surface underfoot. We hypothesized that participants would prefer to reduce their hopping frequency and increase their period of ground contact when hopping on a lower stiffness surface. Second, we aimed to explore the neuromechanical factors that might underpin hopping frequency preference. To achieve this, we measured kinematics, ground reaction forces, and muscle activation of the right lower limb and foot during a bilateral hopping protocol at prescribed and preferred frequencies on elastic and stiff surfaces.

## 2 Methods

### 2.1 Participants

Fifteen healthy participants (5 females, 10 males; age, 27 ± 4 years.; height, 170 ± 8 cm; mass, 73 ± 15 kg) volunteered to participate in this study. All participants were free from lower‐limb injury in the 6 months prior to data collection. The experimental procedures were approved by the local ethics review board at The University of Queensland (IRB:2020/HE000456) and performed in accordance with the declaration of Helsinki.

### 2.2 Experimental Protocol

Prior to data collection, participants were instructed to familiarize themselves with two surface conditions; a control high stiffness surface (Locked), and an elastic low stiffness surface (Elastic). To manage any learning effect, participants completed 30 s of bilateral hopping at four prescribed frequencies on each surface prior to data collection. The frequency settings were as follows: 2.2 Hz (as per prior studies), participants' preferred rate, +20% of their preferred rate (High), and −20% of their preferred rate (Low). Participants were given a period of familiarization to each surface where they were asked to pick a “preferred frequency that you deem most comfortable to sustain”. This frequency was used as participants' “preferred” frequency and was performed first on each surface. Although the exact time to determine preferred frequency for each participant was not documented, Raburn et al. [16] showed that frequency adjustments plateaued within 30 s, as was allowed here. The subsequent trials were then performed in a counter‐balanced order and frequency was matched to the beat of a digital metronome. Collection for each frequency constrained trial was commenced once it was visually deemed that participants were timing their hops to the metronome's beat. For the unconstrained, preferred conditions, participants were instructed that collection would commence once they had informed the researcher when they felt as though they had reached a ‘comfortable rate’. Participants were unshod for all conditions and provided with sufficient rest after each condition that they felt fully ready to complete another trial.

### 2.3 Platform Characteristics

**FIGURE 1:**
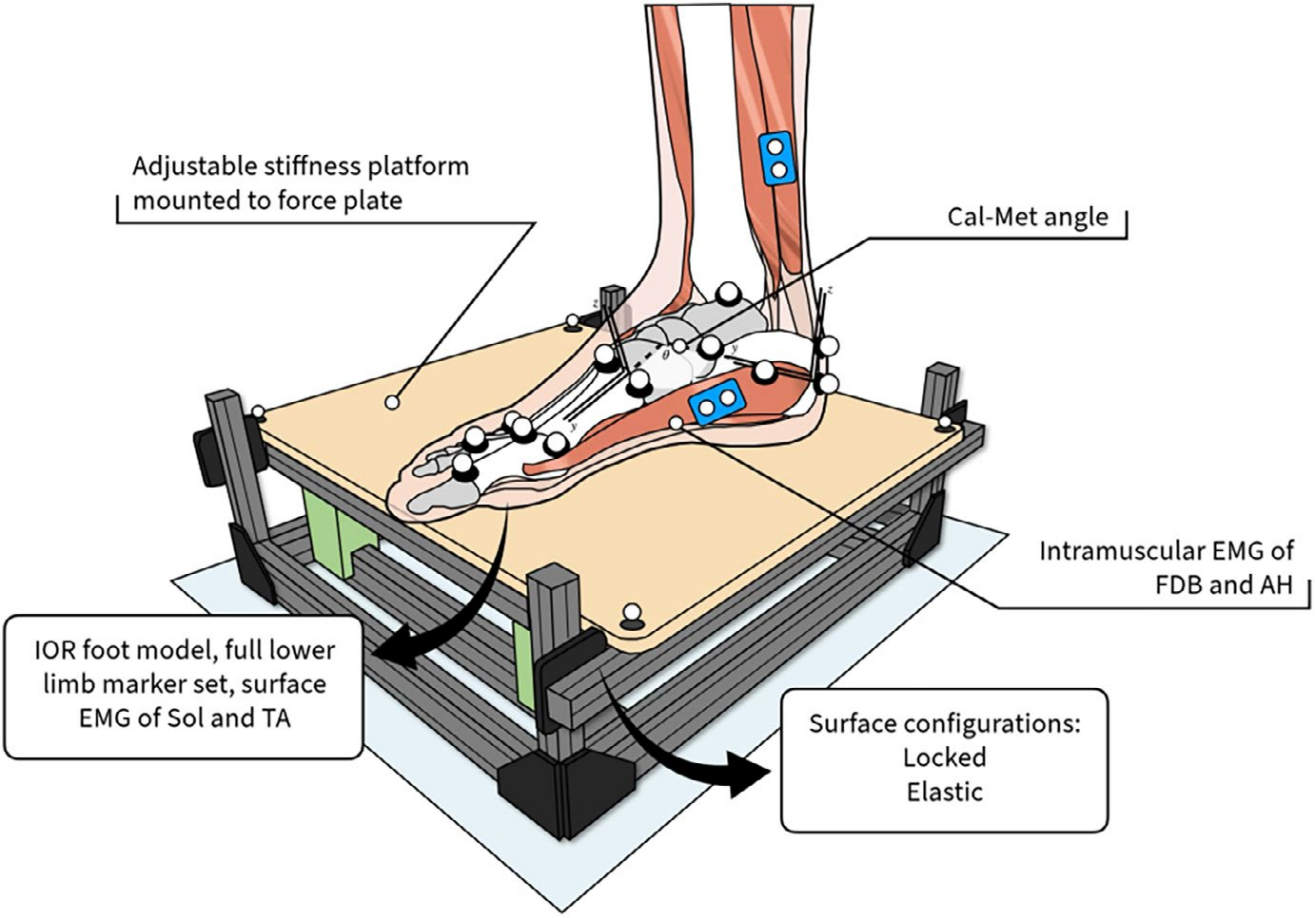
A schematic of one of the platforms, the motion capture markers on the foot, and the EMG electrode locations. The platform surface could be locked or made elastic by the spring‐dampers located underneath (green blocks).

We used an adjustable stiffness surface to manually alter the mechanical properties of the hopping surface (Figure 1). Because we were interested in recording only the ground reaction forces from the right foot in all conditions, two adjustable compression‐sprung platforms were used, with the platform under participants' right foot mounted to an AMTI force plate (OR6‐7; AMTI, Massachusetts). Each platform had identical mechanical properties, only differing in placement within the capture volume. Both platforms featured an extruded aluminum frame with parallel arranged compression springs and four linear sliding bearings in each corner to stabilize the upper surface (as illustrated in [17]). The displacement of the upper surface was tracked using motion capture, and along with ground reaction forces recorded during static loading tests, was used to quantify surface stiffness for the elastic stiffness condition (92 kN.m^−1^). For the Locked surface condition, the compression springs were removed and the upper surface locked to prevent vertical displacement. The resting height of the upper surface was the same for both surfaces. Energy stored in the surface during elastic trials was calculated as the integral of vertical force (measured via force plates under the platform) and surface displacement. Making the assumption that variation in hysteresis would be minimal between conditions, energy stored was considered to correlate with energy returned.

### 2.4 Data Acquisition

#### 2.4.1 Kinematic and Kinetic Measurements

The position of retro‐reflective markers positioned over anatomical landmarks on the right shank and foot of participants was tracked at 200 Hz using an optical motion capture system (Qualisys AB, Gothenburg, Sweden). Foot markers were positioned in accordance with the Istituto Ortopedico Rizzoli (IOR) foot model [18]. To minimize unwanted artifact, markers were adhered using adhesive spray and double‐sided tape, and where possible, further secured with cohesive bandage. Motion data were gen‐locked using an external analogue synchronization pulse from the ADC unit that also synchronously sampled the ground reaction forces and electromyography (EMG) data.

#### 2.4.2 Electromyography (EMG) Measurements

Bipolar, fine‐wire intramuscular electrodes (0.051 mm, stainless steel, Teflon coated; Chalgren Enterprises, California) were inserted into the belly of abductor hallucis (AH) and flexor digitorum brevis (FDB) in the right foot of each participant under sterile conditions and in accordance with previously described B‐mode ultrasound‐guided insertion techniques [14]. Ag/AgCl surface electrodes (Covidien LLC., Massachusetts) were placed over the muscle belly of the right leg's soleus (Sol) and tibialis anterior (TA) muscles to record surface EMG data. All EMG channels were sampled at 4000 Hz, amplified 1000 times, hardware filtered with a bandwidth of 20–2000 Hz, and grounded with a reference electrode placed over the tibial tuberosity. Preamplifiers and cabling were secured using cohesive bandage to prevent signal noise artifacts. Upon visual inspection of poor signal quality, data from one participant were discarded from analyses.

### 2.5 Data Analysis

#### 2.5.1 Kinematics and Kinetics

Marker position data were digitally filtered with a 10 Hz recursive second‐order low‐pass Butterworth filter and used to define and scale a rigid body model of the shank, calcaneus, midfoot, metatarsal, and hallux segments for each participant. From this, six degrees of freedom representations of the metatarsal‐phalangeal joint (MTPj), midfoot, and ankle could be determined. Sagittal plane motion recorded using this approach shows good agreement with segment positions recorded using biplanar video radiography [15]. The orientation of the hallux relative to the metatarsal segment was used to calculate the angle of the MTPj. We computed the midfoot as the orientation of the metatarsal segment with respect to the calcaneus (Cal‐Met angle) with a positive change in the angle representing dorsiflexion of the metatarsals relative to the calcaneus, resulting in compression of the long arch [17]. The ankle angle was computed as the orientation of the calcaneus relative to the shank as per recent recommendations [15, 19]. Joint moments were calculated in Visual3D using an inverse dynamics solution. An assumption was made that the lightweight construction of the platform surface (carbon fiber and aluminum parts) meant that its inertial contributions to contact forces were negligible. Quasi‐stiffness of the ankle and midfoot was calculated as the ratio of the change in moment about each joint to its angular displacement over the period of loading following ground contact (rising moment values). Ground reaction forces were digitally filtered with a 35 Hz recursive second‐order low‐pass Butterworth filter and, using a vertical threshold of 50 N, used to locate the start and end of each hop cycle. The displacement of the body COM during each hop was calculated by twice integrating COM acceleration obtained from force data. Leg stiffness was calculated as the ratio of the peak vertical ground reaction force to the change in length of the leg spring during contact. The resting length of the leg spring was defined as the distance between markers located on the anterior and posterior superior iliac spine and first, second, and fifth metatarsal heads at the instance of each initial ground contact. Data were then exported to MATLAB (The Mathworks Inc., MA, United States) for subsequent analyses.

#### 2.5.2 EMG

Following DC offset removal, all EMG signals were digitally filtered with bi‐directional Butterworth filters. Intramuscular channels were high‐pass filtered at 35 Hz, and surface channels were band‐pass filtered between 35 to 400 Hz. The 35 Hz cut‐off is higher than usual for surface EMG but provided consistency across all signals in our attempt to remove motion artifacts caused by repeat impacts during hopping. EMG envelopes of the resultant signals were generated by calculating the root mean square (RMS) amplitude over a moving window of 50 ms and normalized to the maximum amplitude recorded for the respective muscle during the 2.2 Hz frequency condition on the Locked surface. The normalized RMS envelopes were then integrated (iEMG) with respect to time for the contact phase (iEMG_contact_).

### 2.6 Statistics

Linear mixed models were used to test how humans adapt to frequency and surface constraints. A model was fit for each variable using the MATLAB (The Mathworks Inc., MA, United States) function *fitlme*. The model included fixed effects of frequency, surface, and frequency*surface, and random effects of participant and frequency (to account for between‐participant variability). Post hoc paired *t*‐tests were performed to identify significance between levels of frequency and surface conditions, respectively. An alpha level of *p <* 0.05 was used to determine statistical significance. Results are presented as mean ± standard deviation (SD) unless otherwise stated.

## 3 Results

### 3.1 Hz Versus Preferred Frequency

The frequency selected by participants in the Preferred condition was not different from the 2.2 Hz condition (2.3 ± 0.2 Hz, Table 1, *p* = 0.376). Participants hopped using similar ground contact times (Table 1, *p* = 0.252) and maintained the same vertical COM excursion (Table 1, *p* = 0.313) at both Preferred and 2.2 Hz frequencies, landing with the same kinematic pose at ground contact and generating the same ankle and midfoot work (Table 2). While participants harnessed marginally more energy from the elastic surface when hopping at their preferred frequency (8.4 ± 2 J/hop at preferred compared to 8.0 ± 2 J/hop at 2.2 Hz, Table 1), we detected no difference in any of our outcome variables between preferred and 2.2 Hz conditions on either surface (*p* > 0.05).

**TABLE 1:** Group mean global hopping parameters (mean ± SD, *n* = 12). *p*‐Values are for main effects and interactions in the mixed effects models.

Surface condition	Frequency condition	Actual frequency (Hz)	Contact time (ms)	COM excursion (cm)	Energy stored in platform (J/hop)
Locked	2.2 Hz	2.2 ± 0.1	268 ± 37	11 ± 2	—
	Low	1.9 ± 0.2	322 ± 66	15 ± 3	—
	Preferred	2.3 ± 0.2	257 ± 37	11 ± 1	—
	High	2.8 ± 0.2	216 ± 29	8 ± 2	—
Elastic	2.2 Hz	2.2 ± 0.1	281 ± 48	11 ± 1	8.0 ± 2
	Low	1.8 ± 0.2	351 ± 91	14 ± 4	5.3 ± 3
	Preferred	2.2 ± 0.2	275 ± 52	12 ± 1	8.4 ± 2
	High	2.6 ± 0.2	229 ± 33	8 ± 2	7.3 ± 2
*p*‐value (surface)			< 0.000*	0.346	
*p*‐value (frequency)			0.002*	< 0.000*	
*p*‐value (surface*frequency)			0.002*	0.135	

*Note:* *indicates the criteria for statistical significance was met (*p* < 0.05).

**TABLE 2:** Mean ± SD, ankle and midfoot joint kinematics and kinetics for the Locked and Elastic surfaces (*n* = 12). *p*‐Values are for main effects and interactions in the mixed effects models.

	Surface condition	Frequency condition	Angle at contact (degrees)	Excursion during loading (degrees)	Peak torque (Nm/kg)	Positive work (J/kg/hop)	Joint quasi‐stiffness (Nm/kg/deg)
Ankle	Locked	2.2 Hz	10.6 ± 4.74	22.3 ± 2.48	2.87 ± 0.42	0.47 ± 0.07	0.12 ± 0.02
		Low	13.3 ± 5.85	27.8 ± 4.58	2.53 ± 0.47	0.51 ± 0.10	0.09 ± 0.02
		Preferred	9.53 ± 5.77	20.4 ± 3.69	3.04 ± 0.48	0.46 ± 0.08	0.14 ± 0.03
		High	8.60 ± 5.23	13.1 ± 2.26	2.96 ± 0.46	0.33 ± 0.08	0.23 ± 0.08
	Elastic	2.2 Hz	8.73 ± 5.89	18.8 ± 1.94	2.72 ± 0.42	0.38 ± 0.05	0.15 ± 0.03
		Low	11.3 ± 5.58	25.4 ± 4.31	2.32 ± 0.61	0.44 ± 0.11	0.10 ± 0.03
		Preferred	9.09 ± 5.44	17.9 ± 4.45	2.75 ± 0.39	0.37 ± 0.07	0.17 ± 0.06
		High	7.82 ± 7.05	10.7 ± 3.56	2.76 ± 0.28	0.27 ± 0.06	0.31 ± 0.16
*p*‐value (surface)			0.032*	< 0.000*	0.004*	< 0.000*	< 0.000*
*p*‐value (frequency)			0.009*	0.041*	0.006*	< 0.000*	0.003*
*p*‐value (surface*frequency)			0.756	0.470	0.321	0.718	< 0.000*
Midfoot	Locked	2.2 Hz	−40.0 ± 6.61	16.3 ± 3.46	1.22 ± 0.34	0.12 ± 0.05	0.08 ± 0.03
		Low	−41.0 ± 5.55	17.4 ± 3.60	1.07 ± 0.32	0.12 ± 0.11	0.06 ± 0.02
		Preferred	−40.1 ± 6.54	15.7 ± 3.75	1.19 ± 0.33	0.12 ± 0.11	0.08 ± 0.03
		High	−38.4 ± 7.07	13.4 ± 7.07	1.19 ± 0.36	0.10 ± 0.06	0.10 ± 0.04
	Elastic	2.2 Hz	−39.5 ± 7.80	14.6 ± 2.82	1.09 ± 0.40	0.10 ± 0.05	0.08 ± 0.04
		Low	−40.0 ± 6.11	15.4 ± 2.18	0.85 ± 0.38	0.09 ± 0.11	0.06 ± 0.03
		Preferred	−38.9 ± 6.82	14.2 ± 3.44	1.16 ± 0.37	0.10 ± 0.07	0.09 ± 0.03
		High	−38.8 ± 7.88	12.0 ± 2.94	1.10 ± 0.40	0.09 ± 0.06	0.10 ± 0.03
*p*‐value (surface)			0.079	< 0.000*	0.030*	0.002*	0.059
*p*‐value (frequency)			0.074	0.379	0.522	0.003*	< 0.000*
*p*‐value (surface*frequency)			0.180	0.243	0.152	0.032*	0.077

*Note:* *indicates the criteria for statistical significance was met (*p* < 0.05).

### 3.2 Effects of High and Low Frequencies on Locked and Elastic Surfaces

There was no difference in the vertical excursion of the COM between the elastic and locked surface conditions, despite an increase in contact time on the elastic surface (Table 1). There was a significant effect of frequency on COM excursion, with participants increasing the excursion of their COM with decreasing frequency (Table 1).

### 3.3 Ankle Joint Mechanics

**FIGURE 2:**
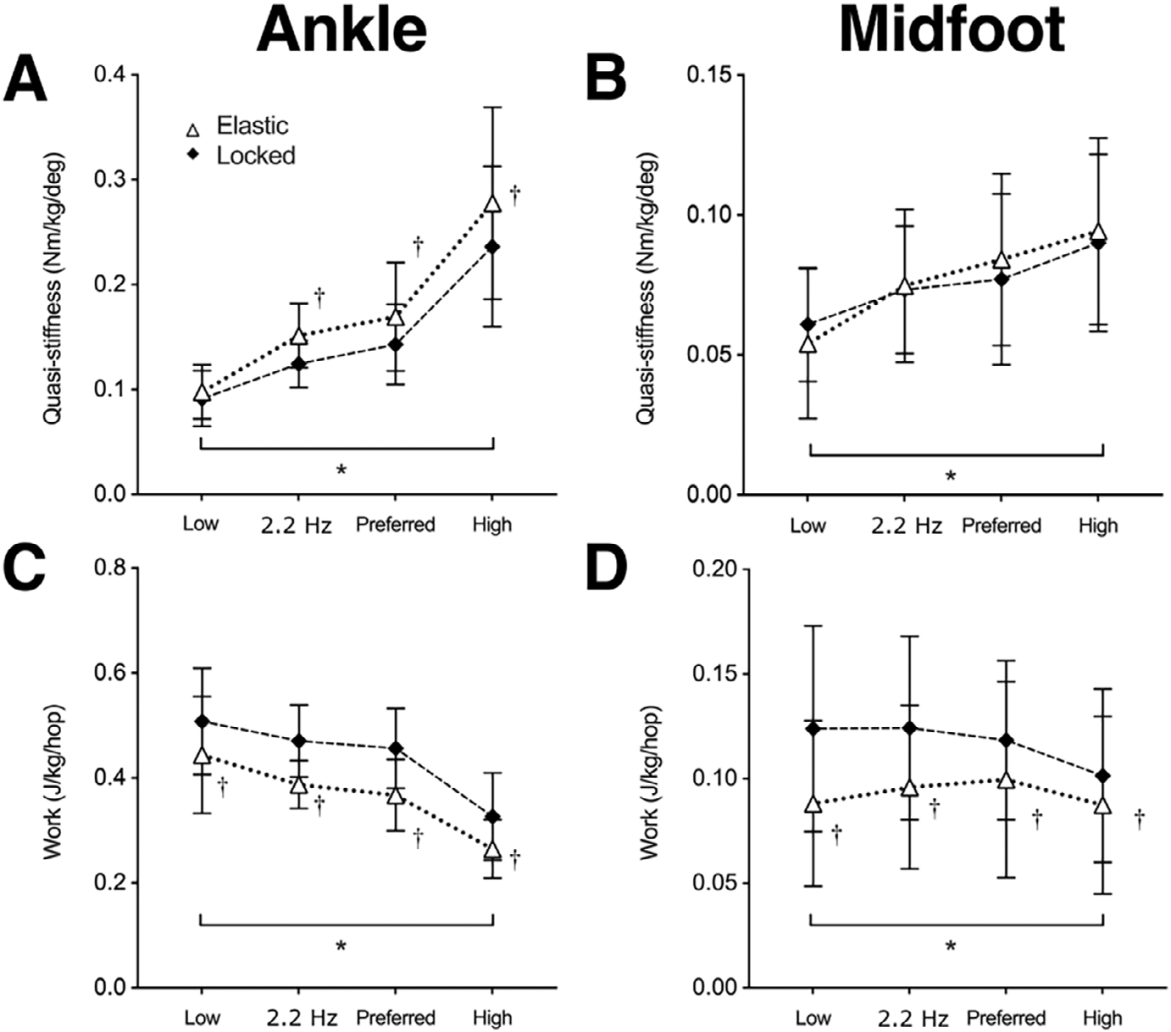
(A, B) Group mean (± SD) quasi‐stiffness of the ankle (A) and midfoot (B) during the loading phase of ground contact for all combinations of surface stiffness and hopping frequency. (C, D) positive mechanical work done at the ankle (C) and midfoot (D) joints during the ground contact phase of hopping. *indicates a significant main effect of frequency; †significant effect of surface at that frequency.

We observed significant effects of surface and frequency on ankle angle at contact (*p* = 0.032, *p* = 0.009; Table 2), ankle excursion during loading (*p* = < 0.000, *p* = 0.041; Table 2), and peak ankle torque (*p* = 0.004, *p* = 0.006; Table 2). Participants were more plantarflexed at contact and went through a greater ankle joint excursion on the locked surface, while also generating greater peak moments in that condition (Table 2). Increasing hop frequencies led to less plantarflexed contact angles, lower excursions, and larger peak torques (Table 2). Participants generated more positive work about their ankles on the locked surface and in the low frequency condition vs. the elastic surface and high frequency conditions, respectively (*p* = < 0.000, Table 2). There was a surface‐by‐frequency interaction effect on ankle quasi‐stiffness, with participants stiffening their ankles to a greater degree on the elastic surface in the high frequency condition (Figure 2).

### 3.4 Foot Mechanics

We observed significant reductions in midfoot excursion (*p* < 0.000) during loading and peak midfoot torque (*p* = 0.030) on the elastic surface (Table 2). Increasing frequency significantly increased midfoot quasi‐stiffness (*p* < 0.000, Table 2). We also observed significant effects of surface, frequency, and surface‐by‐frequency interaction on positive work generated about the midfoot (Table 2). Midfoot work was less on the elastic surface and greatest at preferred frequencies (Table 2). The interaction showed that midfoot work was greatest at low frequency on the locked surface (Table 2).

### 3.5 Muscle Activation

**FIGURE 3:**
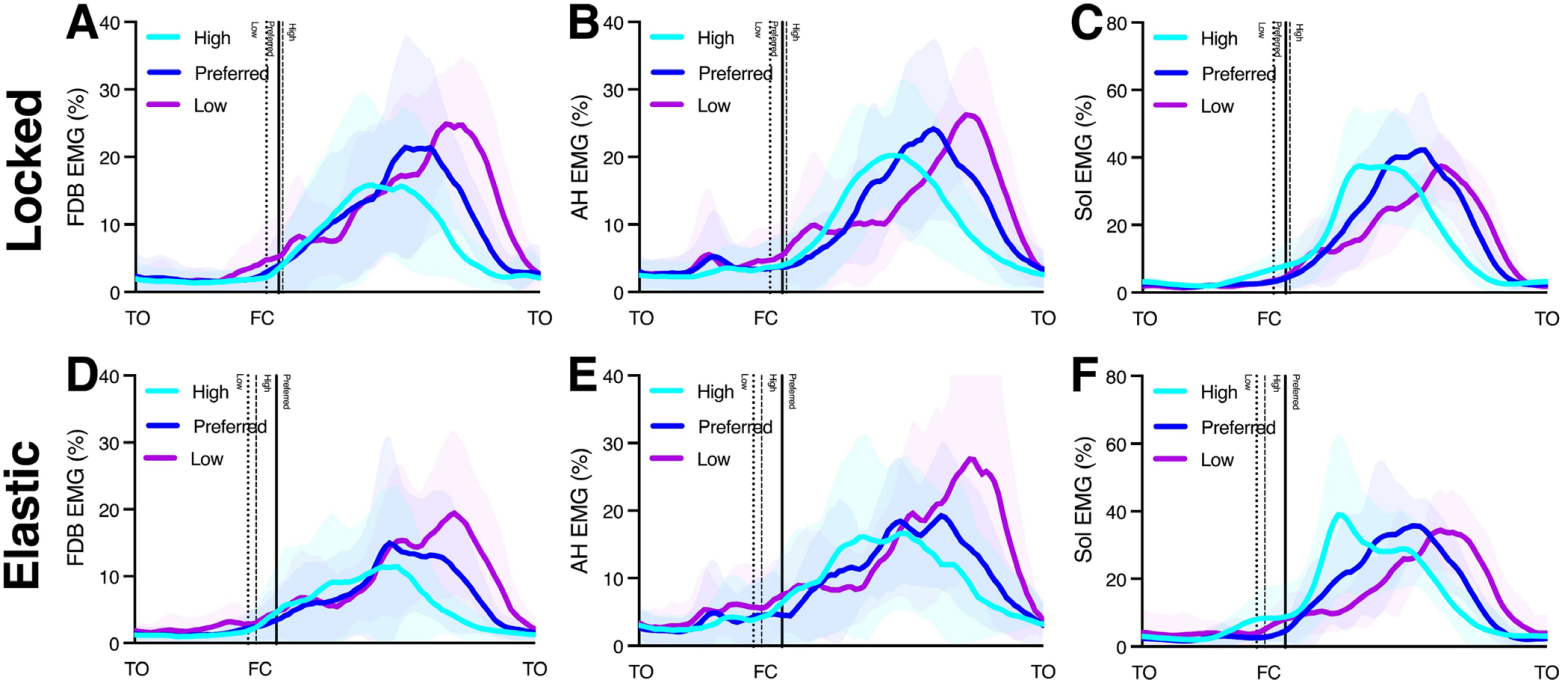
Group mean (± SD) normalized EMG envelopes from preferred, low, and high frequency conditions, plotted against normalized hop cycle time from toe‐off (TO) to toe‐off. The top row (A–C) are for the Locked condition and the bottom row (D–F) the elastic condition. From left‐to‐right, the data are for FDB (A, D), AH (B, E), and Sol (C, F) muscles. Vertical lines indicate the respective average instances of foot contact (FC) for each frequency condition (solid line: preferred; dashed line: high; dotted line: low).

Sol, AH, and FDB muscles displayed similar overall activity with increasing integrated EMG with decreasing frequency on the elastic surface (Table 3). There was a period of inactivity when participants were not in contact with the platforms, followed by a burst of activity during contact (Figure 3). We detected main effects of surface on integrated EMG during contact for FDB, AH, and SOL, showing greater integrated EMG on the locked surface (Table 3). There were also significant main effects of frequency on integrated EMG, with preferred frequencies having the lowest values (Table 3).

**TABLE 3:** Unnormalized group mean integrated EMG values for the period of ground contact (mean ± SD, *n* = 12).

Surface condition	Frequency condition	AH	FDB	Soleus
Locked	2.2 Hz	10.8 ± 1.8	10.7 ± 1.8	10.7 ± 1.8
	Low	12.3 ± 2.2	12.3 ± 2.2	12.2 ± 2.0
	Preferred	11.6 ± 2.7	11.6 ± 2.7	11.5 ± 2.6
	High	9.04 ± 2.1	9.0 ± 2.1	8.9 ± 1.9
Elastic	2.2 Hz	10.0 ± 1.8	10.0 ± 1.8	10.2 ± 1.9
	Low	11.8 ± 1.3	11.8 ± 1.3	12.1 ± 1.6
	Preferred	10.1 ± 1.9	10.1 ± 1.9	10.4 ± 2.1
	High	9.1 ± 1.3	9.1 ± 1.3	9.4 ± 1.6
*p*‐value (surface)		0.015*	< 0.016*	0.030*
*p*‐value (frequency)		< 0.000*	0.000*	< 0.000*
*p*‐value (surface*frequency)		0.744	0.753	0.619

*Note:* *indicates the criteria for statistical significance was met (*p* < 0.05).

## 4 Discussion

### 4.1 Preferred Hopping Frequency Versus 2.2 Hz

To address our first aim, we compared preferred and 2.2 Hz frequency hopping on a locked and an elastic surface. Previously, an average “preferred” frequency of 2.2 Hz has been imposed to understand how humans tune their lower limb mechanics to sustain movement across surfaces with different mechanical properties [4]. Our hypothesis was that participants' preferred frequency would change for an elastic surface, to one that helps minimize muscular work. In contrast to our hypothesis, participants did not alter their behavior when given free choice of frequency and hopped at a frequency that was not significantly different from 2.2 Hz. Participants used similar ground contact times and landing kinematics, resulting in similar motion of their COM for the preferred and 2.2 Hz frequency conditions on each surface (Table 1). Furthermore, participants produced a similar amount of work at the foot and ankle in the preferred and 2.2 Hz conditions, and did so with similar foot and ankle muscle activation levels. Thus, we conclude that, for the range of stiffnesses used, imposing a frequency of 2.2 Hz produces the same hopping mechanics as allowing participants to freely choose hopping frequency. Therefore, prior work that has assumed 2.2 Hz hopping to be representative of preferred hopping has made a reasonable assumption that has not influenced the conclusions. These findings add to prior observations of human's preferred hopping rate [12] that have been used to inform preferred frequency for several subsequent investigations [4, 5, 13, 20, 21]. While this is not what we expected, our finding may help to simplify the design of future studies seeking to understand how humans interact with varied surface types.

It is important to note, however, that an average preferred frequency may not represent the preferred individual frequency for a given participant. There was an interaction effect of surface‐by‐frequency on energy stored in the platform (Table 1) that meant participants harnessed slightly (~ 5%, 0.4 J) more energy per hop from the elastic surface at their preferred frequency compared to 2.2 Hz. This could be linked to participants' COM excursion being marginally greater at preferred frequency on the elastic surface (Table 1) without requiring additional work to be generated by the musculoskeletal system. Therefore, it may be that individual tuning of hopping frequency rather than a systematic shift in preferred frequency allowed participants to maximize the assistance that they receive from the elastic surface (Cavagna and Legramandi 2015). However, this needs to be tested across a wider range of stiffnesses.

### 4.2 Adjustments Made for Elastic Surfaces

For all frequencies, the strategy used by participants on the elastic compared to the locked surface involved storage and return of energy in the platform and reducing the demand for biological tissues of their feet and ankles to contribute to mechanical work. To achieve this, on the elastic surface our participants landed in a more dorsiflexed ankle position and exhibited a reduction in joint excursions in the midfoot and ankle (Table 2). The lesser joint excursions contributed to reduced midfoot and ankle positive work, and greater ankle and midfoot quasi‐stiffness values (Table 2). The latter being despite a reduction in peak ankle and midfoot torques and muscle activations for the elastic surface (Table 2 and Figure 3), as well as longer contact times (Table 2). These findings from the present work reproduce well the results of our prior study on bilateral hopping on elastic surfaces [9]. The overall response to the reduced in‐series stiffness of the elastic platform seems to be to produce a stiffer behavior of the foot and ankle and allow energy to be stored and returned in the surface while reducing neuromuscular effort (quantified by muscle activation and positive work). This is partially consistent with prior work showing that humans faced with less‐stiff surfaces will stiffen the lower limb to maintain overall limb‐surface system stiffness and COM motion [4]. However, the ability to increase ankle and midfoot joint stiffness while concurrently reducing muscle activations and joint torques is somewhat non‐intuitive and suggests that the kinematic posture on the elastic surface is providing some effective mechanical advantage that the more plantarflexed position on the locked surface is not. However, this latter point is speculation that the present data cannot confirm. Our observations do further indicate the important role of the foot in adapting the mechanical response of the lower limb to changing surface demands.

### 4.3 Factors Underlying Hopping Frequency Preferences

To understand the factors that drive human hoppers’ frequency preference and whether this changes on an elastic surface, we imposed high and low frequency conditions 20% either side of participants’ preferred hopping rate on both the locked and an elastic surfaces. We hypothesized that participants’ preference on the elastic surface would be driven by a desire to maximize the energy that they harnessed from the springs incorporated into the platforms. This prediction was supported with respect to the low frequency condition, with participants storing ~60% more energy in the platform for the preferred compared to −20% condition (Table 1). Accordingly, participants' ankle joint work was lower at their preferred frequency compared to the low frequency condition on the elastic surface (Table 2). With less assistance provided by the surface at low frequencies, participants were required to activate their foot and ankle muscles more to generate more mechanical work per hop (Figure 2 and Table 3). The increased ankle joint work at the lowest frequency concurred with reduced ankle quasi‐stiffness and increased ankle joint excursion (Table 2). The latter contributed to greater COM excursion during ground contact, a change that is known to be metabolically costly [22, 23]. At very low hopping frequencies, humans have been shown to be unable to operate in a spring‐like manner and have to recruit more proximal muscle groups (hip and knee extensors) to help generate mechanical work [12], as well as dissipating energy that could otherwise be stored and returned in elastic structures [12]. Our participants did more mechanical work and had to activate their foot and ankle muscles more in the low frequency condition (Figures 2 and 3), presumably incurring a greater energy cost per hop. This finding agrees with prior work describing single leg hopping [15], where increasing activation of the intrinsic foot muscles was linked to the increased mechanical work demands of hopping at low frequencies.

While participants used a lower COM excursion and performed less work at their preferred hopping rate with respect to the low frequency (Table 1), work was not reduced with respect to the high frequency conditions on each surface (Table 2). Consequently, hoppers generated more foot and ankle work at their preferred frequency on each surface than they did in the high frequency conditions (Table 2). Per hop, contact phase activation and work were reduced in the high frequency conditions, which was expected given the reduced mechanical work demands observed here and previously associated with hopping at high frequencies [15]. However, it is important to consider the consequence of participants generating greater torque and quasi‐stiffness (Figure 2) over a shorter period of ground contact (Table 1) on the metabolic cost associated with generating muscular force. The rate at which muscles consume metabolic energy to sustain locomotion increases with movement frequency and the reduced ground contact times that come with it [3]. Despite decreasing muscular work, force cycling in the ankle plantar flexors has been shown to increase with frequency and explains greater metabolic costs during human bouncing and hopping tasks at frequencies above 3 Hz [24, 25]. The effects of maintaining steady state hopping at high frequencies would likely have increased the rate of metabolic energy consumption due to the increased rate of force production (per hop and per second) required [2, 3]. That our participants preferred to hop at an intermediate rate seems to reflect a compromise between minimizing both mechanical work and rate of force production to minimize the energy cost of hopping. We have shown that the neuromechanics of the foot reflect those of the ankle and COM in this regard.

### 4.4 The Interaction Between the Surface and Frequency

While the strategy used to hop showed similar trends with increasing frequency on both surfaces (decreasing activation and COM excursion), there was an interaction effect for surface and frequency on ankle quasi‐stiffness. To make hopping on the elastic surface possible at the high frequency, participants used greater ankle quasi‐stiffness, but not at low frequencies (Figure 2). The reduced ground contact times required to maintain higher frequencies combined with the reduction in surface stiffness likely necessitated greater quasi‐stiffness to preserve their COM excursion. Most interestingly, whereas participants generated more work at their midfoot with decreasing frequency on the locked surface, they produced less work at their midfoot at the low frequency on the elastic surface (Figure 2). By altering the timing of their hop to match the beat of the metronome, participants harnessed less energy from the elastic surface and may not have been able to store and return energy from their feet as effectively. Consequently, they may have recruited more proximal sources of work [12]. The observed interactions of surface stiffness and hopping frequency suggest that we cannot assume similar trends in leg spring mechanics to occur with changing frequency on differently elastic surfaces.

### 4.5 Limitations

The change in stiffness provided by the elastic surface was not as large as those that have been used previously when observing how humans interact with elastic surfaces [4, 5, 9]. Given that we observed non‐significant trends between the 2.2 Hz and preferred frequency conditions, it is possible that greater effects of surface and frequency interactions might be observed if faced with a bigger perturbation in surface stiffness. Larger changes between surfaces, while potentially highlighting changes in preference, may not be relevant to the adaptations used by humans in the real world. Future studies are welcomed to understand if humans tune their preference across a wider range of surface stiffnesses. Additionally, we observed changes in mechanics in this study consistent with a potential reduction in metabolic cost at hopping humans' preferred frequency. Metabolic data across a wider range of frequencies would have helped to uncover if this is the case.

## 5 Perspectives

Our work sought to provide fundamental insights into how the stiffness of the surface under human feet during bouncing gaits influences our movement preferences and explains the outcomes based primarily on foot and ankle neuromechanics. We are constantly faced with the need to adapt to different underfoot stiffness, be it the ground itself or the footwear we choose. Modern high‐performance running shoes with high energy storage and return ratios are an example where modifying elastic properties has benefited sports performance. The present study has shown that, on average, preferred hopping frequency and the gross mechanics of hopping do not change on a less stiff surface, indicating our ability to adapt readily to stiffness changes. We also observed changes in neuromuscular and mechanical behavior at the foot and ankle that highlight the importance of foot and ankle muscles in facilitating the aforementioned adaptation.

## 6 Conclusions

Here we highlighted features of foot and ankle mechanics that are tuned to surface and frequency constraints during bilateral hopping. We found that a 2.2 Hz hopping frequency does represent human hoppers’ average preferred frequency on the range of surface stiffnesses used. Factors driving this frequency preference appear to be maximizing elastic assistance from a surface while trading off the costs of active muscular work and the cost associated with producing force. However, it should be considered that the group average preferred frequency may not be exactly optimal for all individuals, and frequency preference is an important factor for future work. Furthermore, the presence of interaction effects of surface and frequency on hopping mechanics suggests that the effects of frequency vary on differently elastic surfaces. Overall, our findings highlight the important role of the foot in adapting the mechanical response of the lower limb to changing surface demands and frequency.

## Conflicts of Interest

The authors declare no conflicts of interest.

## Data Availability

The data that support the findings of this study are available from the corresponding author upon reasonable request via an online institutional repository.
